# The complete chloroplast genome sequence of the medicinal plant *Cercis chinensis* and phylogenetic analysis

**DOI:** 10.1080/23802359.2021.1999189

**Published:** 2021-11-12

**Authors:** Jiu-Lue Hu, Zhu Hou

**Affiliations:** a Zhang Zhongjing College of Chinese Medicine, Nanyang institute of Technology, Nanyang, China; b Henan Key Laboratory of Zhang, Zhongjing Formulae and Herbs for Immunoregulation, Nanyang, China; c China West Normal University, Nanchong, China

**Keywords:** *Cercis chinensis*, chloroplast genome, phylogenetic analysis

## Abstract

*Cercis chinensis* is a deciduous shrub or small landscape tree with exceptional ornamental characteristics. Here, we report the complete chloroplast genome of *C. chinensis* to provide a foundation for further phylogenetic studies of the Leguminosae. The chloroplast (cp) genome was 158,999 bp in size containing a large single-copy (LSC) region 88,176 bp in length, a small single-copy (SSC) region 19,583 bp in length, and two inverted repeat (IR) regions that were 25,620 bp each. The total GC content of the cp genome was 42.9% with the LSC, SSC, and IR regions 36.2, 33.8, and 29.4%, respectively. The cp genome contains 129 genes, including 85 protein-coding, 36 tRNA, and 8 rRNA genes. The phylogenetic analysis revealed that *C. chinensis* is closely related to *Cercis glabra*. These results provide valuable information about the evolutionary processes and conservation genetics of *C. chinensis*.

*Cercis chinensis* (Bunge 1966) belongs to the Leguminosae family (Davis et al. 2002). They are deciduous shrubs or small landscape trees, which exhibit considerable morphological diversity (Roberts et al. 2015). *Cercis chinensis* is widely cultivated in botanical gardens and parks throughout China (Li et al. 2017). For a better understanding of the relationships of *C. chinensis* and other *Cercis* species, it is necessary to reconstruct a phylogenetic tree based on high-throughput sequencing approaches. In this study, we reported and characterized the complete chloroplast (cp) genome of *C. chinensis* based on the Illumina pair-end sequencing and compared it with other cp genomes within the genus. The results of this study provide valuable information for the evolutionary processes and conservation genetics of *C. chinensis*.

*Cercis chinensis* samples were collected from Nanchong, Sichuan province, China (106°08′E; 30°79′N). A specimen was deposited at the NanYang Medical College herbarium, Department of Chinese Medicine (http://www.nymc.edu.cn/, Jiu-lue Hu, tianxtpljh2002@163.com), under the voucher number ZJ001. Total genomic DNA was extracted from fresh and healthy leaves with the DNA Secure Plant Kit (Tiangen Biotech, Beijing, China) following the manufacturer’s protocol. The DNA samples were stored at −80 °C at the Key Laboratory of the Department of Chinese Medicine, Nanyang, China. After library preparation, the DNA library was sequenced on the Illumina HiSeq 4000 sequencing system (Illumina, San Diego, California, USA). We obtained a total of 5.4 Gb raw reads. The raw sequence data were deposited into the NCBI SRA (project accession SRR14793490). The raw data were further filtered using Trimmomatic Version 0.32 with default settings (Bolger et al. 2014). The remaining clean reads were used to assemble the cp chloroplast genome using SPAdes v.3.9.0 (Nurk et al. 2017). The final assembled sequence was annotated with MPI-MP CHLOROBOX (https://chlorobox.mpimp-golm.mpg.de/geseq.html) via GeSeq. The complete *Cercis glabra* cp genome (NC036762) was used as the reference and then corrected using Geneious Prime v2020.2 (Kearse et al. 2012). Finally, the complete chloroplast genome of *C. chinensis* was submitted to GenBank (Accession No. MZ128523).

The cp genome was 158,999 bp in size and consisted of a large single-copy (LSC) region 88,176 bp in length, a small single-copy (SSC) region 19,583 bp in length, and two inverted repeat (IR) regions each 25,620 bp in length. The total GC content of the cp genome was 42.9% with the LSC, SSC, and IR regions 36.2, 33.8, and 29.4%, respectively. The cp genome contained 129 genes, including 85 protein-coding, 36 tRNA, and 8 rRNA genes.

To further investigate its taxonomic status, a maximum likelihood (ML) tree was constructed based on complete chloroplast genome sequences using MEGA 7.0 (Kumar et al. 2016) with 1,000 bootstrap replicates. The program operating parameters were set as follows: a Tamura 3-parameter (T92) nucleotide substitution model with 1,000 bootstrap repetitions, accompanied by Gamma distributed with Invariant site (G + I) rates, and partial deletion of gaps/missing data. Complete cp genome sequences of 14 other Fabaceae species and *Duparquetia orchidacea* as the outgroup were downloaded from GenBank. The 16 chloroplast genome sequences were first aligned with MAFFT (Katoh and Standley 2013). The phylogenetic relationships of these species in relation to the *C. chinensis* cp genome were reconstructed using maximum likelihood (ML) (Figure 1). The phylogenetic analysis revealed that *C. chinensis* was closely related to *Cercis glabra*. These results provide valuable information into the evolutionary processes and conservation genetics of *C. chinensis*.

**Figure 1.**
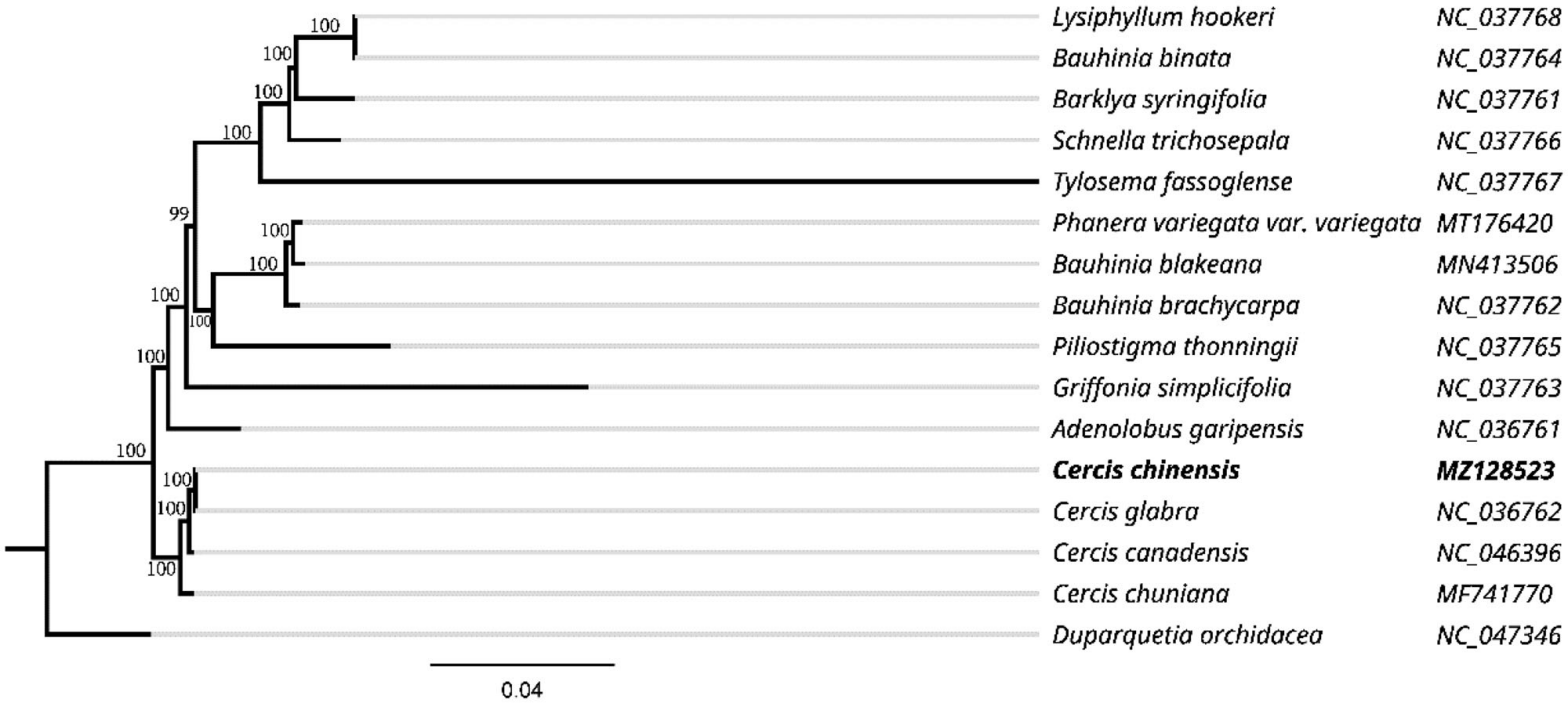
Maximum likelihood phylogenetic tree of *Cercis chinensis* and other related species based on the complete chloroplast genome sequence.

## Data Availability

The genome sequence data that support the findings of this study are openly available in GenBank of NCBI at (https://www.ncbi.nlm.nih.gov/) under the accession No. MZ128523. The associated BioProject, SRA, and Bio-Sample numbers are PRJNA737038, SRR14793490, and SAMN19678361 respectively.
